# Genetic Code Expansion Enables Oriented, Site-Specific Conjugation of DARPins to Lipid Nanoparticles for Selective mRNA Delivery to CD8^+^ T Cells

**DOI:** 10.3390/ijms27177755

**Published:** 2026-08-29

**Authors:** Anastasiia Dakhnevich, Ildus Pateev, Ivan A. Skvortsov, Sofia Yarmachkova, Daniil Shevyrev, Anastasia Chubarova, Mariia Gasina, Nadezhda Cherepanova, Elizaveta D. Siaglova, Vasiliy Reshetnikov, Maksim V. Baranov, Roman A. Ivanov

**Affiliations:** 1 Biotechnology Department, Sirius University of Science and Technology, 354349 Sirius, Russia; 2 Medicinal Chemistry Core Facility, Sirius University of Science and Technology, 354349 Sirius, Russia; 3 Biomaterials Core Facility, Sirius University of Science and Technology, 354349 Sirius, Russia

**Keywords:** lipid nanoparticles (LNP), mRNA, click-chemistry, genetic code expansion, DARPin, CD8 T-cells, cell-targeted gene therapy

## Abstract

Lipid nanoparticles (LNPs) are an effective platform for delivering therapeutic payloads, but their use for targeting specific cell populations in vivo is limited by a lack of specificity. To address this issue, we developed an innovative approach for creating targeted LNPs based on the post-insertion of protein–lipid conjugates. By expanding the genetic code, a single p-azido-L-phenylalanine residue was specifically incorporated into a DARPin against murine CD8, enabling copper-free click-chemistry with dibenzocyclooctyne (DBCO) to functionalize the particle surface with DARPin conjugates. DARPin-LNP characterization demonstrated preservation of particle size and acquisition of a characteristic negative charge after DARPin insertion, confirming successful post-insertion. In vitro experiments using primary murine splenic T-cells showed that targeted LNPs selectively transfected CD8^+^ T-lymphocytes. These data indicate that the developed platform enables selective in vitro delivery of nucleic acids to cytotoxic lymphocytes, highlighting its potential relevance for designing safer immunotherapeutic strategies.

## 1. Introduction

Nanoparticles offer numerous advantages as drug carriers, including nanoscale size, high surface-to-volume ratio, potential for selective targeting, and controlled drug release. LNP systems offer a biocompatible delivery method and are rapidly being adopted in RNA therapy [1]. The successful use of mRNA-LNPs in vaccines against infectious diseases has opened broad prospects for their use in cancer immunotherapy and genome editing [2]. The main advantages of LNP as a non-viral delivery system include lower immunogenicity, no risk of viral genome integration, and enhanced biocompatibility [3,4,5]. However, a key limitation of standard LNPs remains their lack of tissue specificity: when administered systemically, most nanoparticles accumulate nonspecifically in the liver and spleen [6,7]. Modern attempts to alter the biodistribution of LNPs focus on modifying the lipid composition [8,9]. Such changes can affect how LNPs interact with cell membranes and may redirect them toward different cell populations. However, it is difficult to adapt this strategy for targeting a specific cell type. To overcome this limitation, targeted delivery requires modifying or decorating the LNP surface with ligands capable of recognizing specific receptors on target cells. Decorating the surface of lipid nanoparticles with antibodies, antibody fragments, or antibody-like proteins is a key strategy to overcome their nonspecific accumulation in the liver and ensure selective delivery to target organs [10]. Nanoparticles can be functionalized with proteins by adsorption, covalent linkage, or adaptor molecules. When immobilizing antibodies, conjugation must ensure the desired number of biomolecules per nanoparticle and their correct orientation [11].

Traditionally, monoclonal antibodies are used for targeted delivery, but their high molecular weight, complex spatial structure, and high production cost limit their widespread use. This strategy has recently led to the successful in vivo generation of CAR-T cells using LNP-RNA targeting CD5, a marker of T lymphocytes [12]. Scaffold proteins, including artificial proteins, antibody mimetics, and Designed Ankyrin Repeat Proteins (DARPins), are considered an effective alternative to antibodies [13]. DARPins are a class of artificially constructed, small (13–16 kDa), single-domain antibody-mimetic proteins based on natural ankyrin repeats [14]. Due to the absence of disulfide bonds, high thermodynamic stability and high-yield expression in bacterial systems, these proteins hold great promise as ligands for targeted delivery of LNPs, selective targeting of tumor antigens, and the development of diagnostic platforms [13,15]. With their high affinity, low molecular weight, and high stability, DARPin proteins can bind specifically to surface antigens of immune cells, including the murine CD8 (mCD8) marker of mouse T lymphocytes [16].

Ligand binding to the surface of LNPs is most often carried out through covalent conjugation with functionalized polyethylene glycol (PEG) molecules, which are part of the lipid envelope. Traditionally, this has been achieved by randomly modifying the side chains of cysteine (Cys) or lysine (Lys) residues in proteins, but it is becoming increasingly clear that site-specific protein conjugates are often required for certain applications. The most common approach for directed conjugation of proteins with LNP is the formation of a thiol bond between a maleimide group (PEG–maleimide) and a cysteine residue in the protein [17]. Despite the successful use of an FDA-approved immunosuppressant based on the PEGylated thiol–maleimide adduct Cimzia^®^ (certolizumab pegol), similar conjugates in the context of LNP formulation/decoration may have significant limitations. The use of these compounds in vivo may be subject to degradation or transformation by thiol exchange or stabilizing ring opening [18]. The use of this method is associated with the risk of nonspecific binding and low stability of the maleimide linker during conjugation in buffers with pH ≥ 7.5 and in biological media due to hydrolytic cleavage of the maleimide to the corresponding N-maleamic acid derivative [19]. Moreover, strict control of the reduction conditions is required to prevent the formation of disulfide bonds before interaction with the maleimide residue.

As an alternative to conjugation between maleimide on the surface of a particle and cysteine in a protein, bioorthogonal click chemistry, specifically copper-free azide-alkyl cycloaddition (SPAAC), is of increasing interest [20]. Although conventional copper-catalyzed azide–alkyne cycloaddition (CuAAC) is highly efficient, the required Cu(I) species cause severe cytotoxicity and catalyze the formation of reactive oxygen species, leading to oxidative degradation of biomolecules. In contrast, SPAAC circumvents this limitation by exploiting the ring strain of cyclooctynes (e.g., DBCO or BCN) to lower the activation energy barrier, thereby driving a rapid, highly selective dipolar cycloaddition under physiological conditions without the need for transition-metal catalysts. This method requires introducing unnatural functional groups into the protein. We propose a conjugation approach based on the direct incorporation of unnatural amino acids, such as p-azido-L-phenylalanine (pAzF), at a precisely defined position in the protein sequence, enabling strictly site-specific conjugation with PEG lipids modified with dibenzocyclooctyne. CD8^+^ mouse T lymphocytes were selected as a model population of target cells to test this approach to targeted LNP delivery. Cytotoxic CD8^+^ T lymphocytes play a central role in the adaptive antitumor and antiviral immune response by directly lysing transformed cells through the secretion of perforin and granzymes and the expression of the Fas ligand. However, within the tumor microenvironment, these cells undergo progressive dysfunction and metabolic exhaustion, which limits the effectiveness of standard therapies [21]. Targeted delivery of therapeutic nucleic acids using lipid nanoparticles can overcome this barrier by protecting molecules from nuclease degradation and enabling local reprogramming of cytotoxic T cells in vivo with minimal systemic toxicity [22,23]. Modification of LNPs for precision targeting of CD8^+^ T cells or antigen-presenting cells induces antigen-specific proliferation and restores the cytotoxic potential of lymphocytes, which is critically important for overcoming tumor resistance [22]. In addition, targeted delivery of therapeutic mRNA through conjugation of LNPs with anti-CD8 ligands eliminates the expensive ex vivo stage of CAR-T therapy and minimizes systemic hepatotoxicity [24]. The clone MSE10, previously described in the literature, was chosen as a model DARPin [25]. In this work, for the first time, LNP-encapsulated mRNAs were targeted to a specific immune cell population through functionalizing the LNP surface with a DARPin protein containing the unnatural amino acid p-azido-L-phenylalanine, which was introduced into the protein structure through biotechnological synthesis in *E. coli* cells. The resulting particles showed pronounced specificity for CD8^+^ cells in in vitro tests using primary murine splenic T-cells. DARPin, attached to the LNP surface in the correct orientation through a strong click-chemical bond, remains functional, making this approach a new platform technology for precisely targeting RNA-LNPs to specific cell populations.

## 2. Results

### 2.1. Genetic Encoding of the pAzF into DARPin

**Figure 1:**
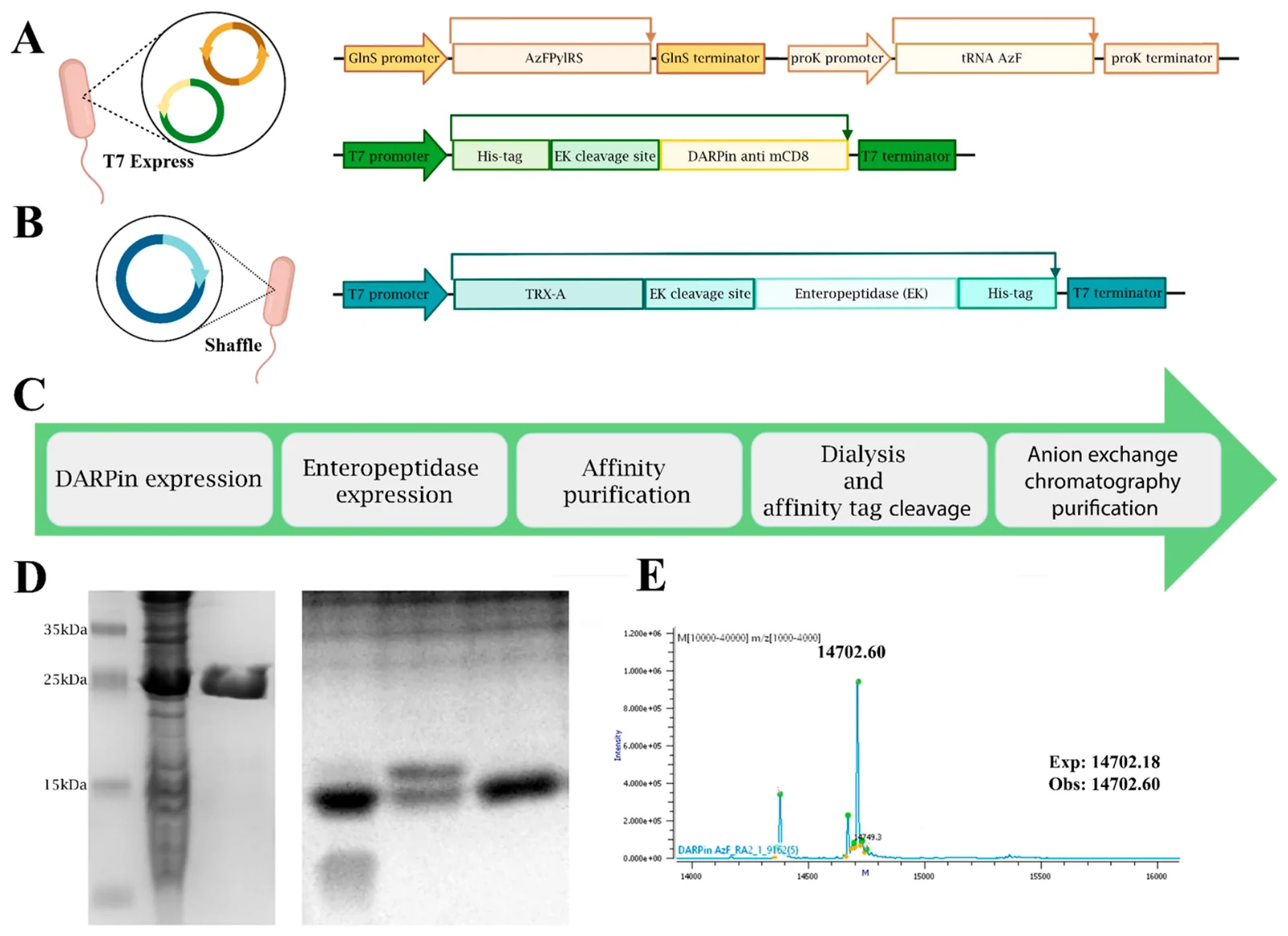
Expression and purification of DARPin with p-azido-L-phenylalanine (DARPin-AzF) targeted to mouse CD8 T-cells. (**A**) Schematic representation of the DARPin-AzF producer strain containing two plasmids, and schematic representation of their open reading frames. (**B**) Schematic representation of a producer strain containing a plasmid for expression of human enteropeptidase light chain (hEKL), and schematic representation of the open reading frame of this plasmid. (**C**) Schematic of the DARPin-AzF bacterial expression process and purification stages with purity assessment by SDS-PAGE. (**D**) SDS-PAGE of purified proteins corresponding to the phases from the expression and purification scheme above. 2—Soluble protein fraction after production; 3—Protein fraction after IMAC purification; 4—Fusion protein after proteolysis; 5—Fractions after anion exchange purification. (**E**) High-resolution electrospray-ionization mass spectra of DARPin-AzF.

For site-specific insertion of pAzF at the C-terminus of anti-murine CD8 DARPin, a flexible linker containing an amber stop codon was added. This construct also contained a His tag at the N-terminus for ease of purification. After isolation of the target protein from the cell lysate and chromatographic purification, the affinity tag remains covalently bound to the protein, which requires its subsequent removal. For this purpose, the proteolytic cleavage site of enteropeptidase, a highly specific protease that cleaves its substrate without leaving amino acid residues at the N-terminal part of the target protein, was cloned between the DARPin and His-tag sequences (Figure 1A).

Site-directed incorporation of ncAA at strictly defined positions in proteins requires appropriate cellular mechanisms. AaRS that do not interact with proteinogenic amino acids and endogenous tRNAs, but can participate in protein synthesis by selectively adding a non-proteinogenic amino acid to the translation product, are called orthogonal [26]. One of the most effective and widely used translation systems for implementing this method is pyrrolysyl tRNA synthetase (PylRS), which aminoacylates its complementary tRNA [27]. A synthetase capable of efficiently incorporating the azide derivative of phenylalanine was developed earlier in the project [28] (Figure 1A).

The expression of DARPin with pAzF was carried out in the *E. coli* T7 Express strain, co-transformed with a plasmid containing the DARPin gene and a plasmid containing AzFPylRS and its corresponding tRNA (Figure 1A). The molecular weight of the fusion protein, calculated from the obtained amino acid sequence, was 19.1 kDa. The size of the fusion protein observed by SDS-PAGE corresponded well to the calculated molecular weight (Figure 1D(2–3)). The protein was purified from *E. coli* lysates. The high purity of the protein was confirmed by SDS-PAGE analysis (Figure 1D(3)). The fusion protein yield was 30 mg/L.

In-house-produced enteropeptidase was used to remove the fusion tags. The human enzyme (hEKL), which has 10 times higher catalytic efficiency (kcat/KM) than the bovine enzyme, was selected for production [29]. Given the nine cysteines in the light chain of enterokinase and the four disulfide bonds in its structure, expression of this protein in active form in the cytoplasm is difficult. We took a number of measures to solve this problem. First, substitution of the free cysteine with serine (C112S) in the amino acid sequence of hEKL helped reduce the number of potential pairs that could form incorrect disulfide bonds, limiting the number of alternative folding pathways and shifting the balance toward the active conformation of the enzyme. Second, to address the insolubility problem, the protein was fused with thioredoxin A (TRX-A) (Figure 1B), which, due to its physicochemical properties, increases the solubility of the expressed protein and promotes the formation of disulfide bonds when expressed in the cytoplasm [30]. SHuffle^®^ T7 Express *E. coli* was chosen as the production strain, as it ensures the formation of S–S bonds due to the oxidizing environment of the cytoplasm [31]. Additionally, transformation was performed using the auxiliary plasmid p.Gro7, adapted to maintain correct folding of proteins with disulfide bonds and containing the GroEL/GroES chaperone complex [32]. When choosing optimal conditions for enteropeptidase production, the development was based on the protocol of Ebrahimifard et al. [33], with a significant modification being the expression temperature at 28 °C.

After affinity chromatographic purification of DARPin anti-mCD8 with pAzF (DARPin-pAzF) with fusion tags, dialysis was performed against a buffer containing calcium chloride, which is necessary for correct enzyme activity of hEKL to obtain free DARPin. The reaction mixture was purified using anion-exchange chromatography, and DARPin purity reached 90% (Figure 1C,D(5)). The yield of the target protein was 25 mg per 1 L of culture.

### 2.2. Validation of SPAAC Protocol with Genetically Encoded pAzF

**Figure 2:**
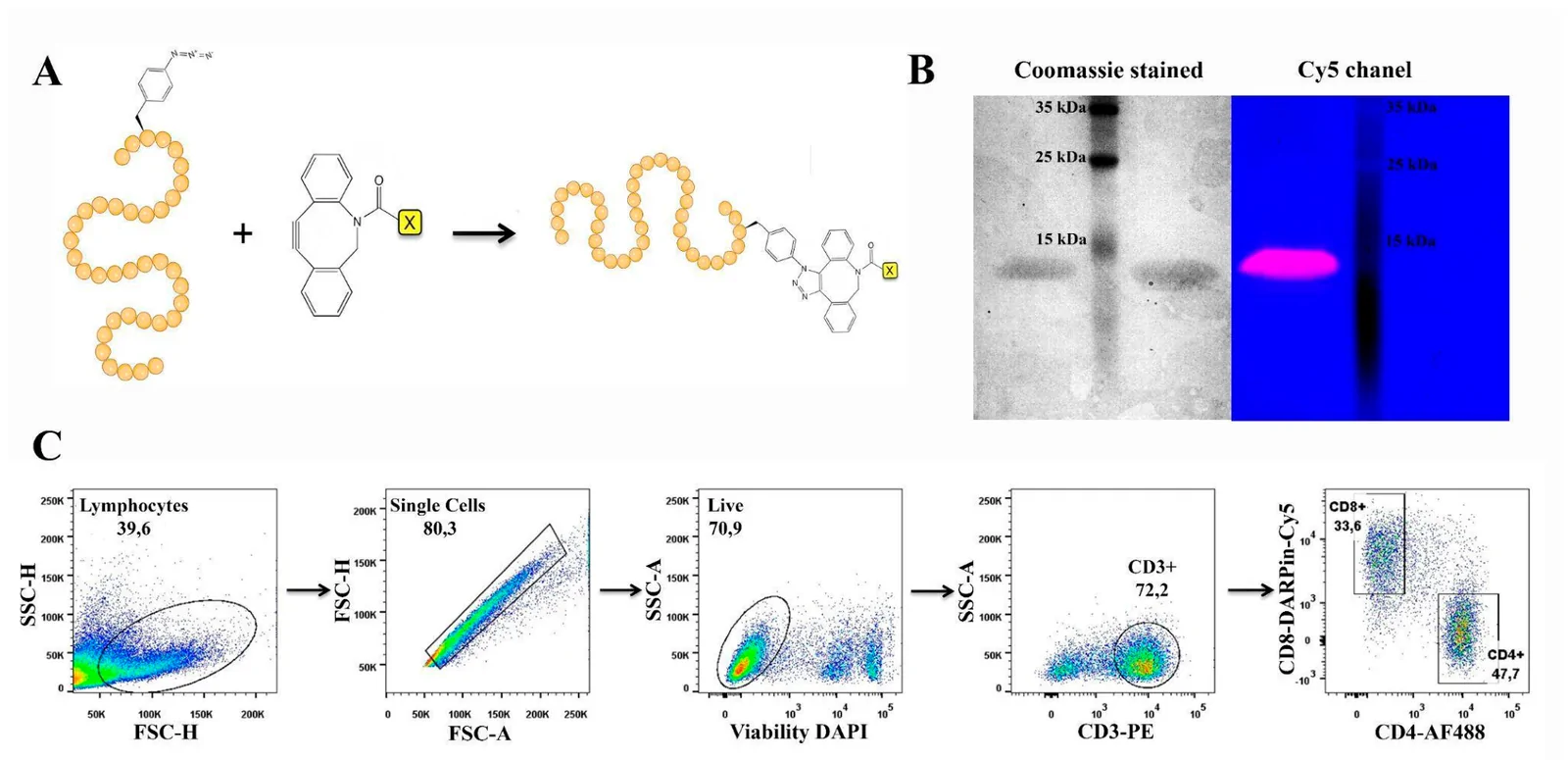
(**A**) Schematic representation of labeling of DARPin-AzF with DBCO-Cy5 via SPAAC reactions. (**B**) SDS–PAGE of products generated by reaction of DARPin-AzF and DARPin-WT with DBCO-Cy5 was imaged by Coomassie staining and in-gel fluorescence. DBCO-Cy5 excitation: 630 nm, emission 650 nm. (**C**) Flow cytometry gating strategy for murine T-cells. After scatter gating and singlet exclusion (FSC-A/FSC-H), viable (DAPI^−^) CD3^+^ T-cells were analyzed for CD4 (commercial Ab) and CD8 expression. CD8 was detected using a home-made Cy5-conjugated DARPin, site-specifically coupled via SPAAC utilizing an AzF-DBCO conjugation strategy.

Cy5-DBCO dye was selected to optimize the bioorthogonal conjugation protocols for DARPin-AzF. SPAAC with dibenzocyclooctyne derivatives is well known to be ideally suited for bioconjugation because it does not require additional reagents and has a high rate constant [34,35]. Using a fluorescent dye as a model substrate for the SPAAC reaction allowed us to optimize the site-specific conjugation conditions and confirm successful incorporation of the noncanonical amino acid by gel electrophoresis before proceeding to more complex functional conjugates (Figure 2A). The conjugation reaction was carried out by adding Cy5-DBCO in 25 mM Tris buffer (pH 7.5) at room temperature overnight. The conjugation reaction was analyzed using SDS-PAGE and fluorescent gel imaging (Figure 2B). When excited at a wavelength of 630 nm, DARPin-Cy5 exhibited characteristic fluorescence at 650 nm. The images obtained from this analysis showed that Cy5-DBCO efficiently conjugated with DARPin. The same conjugation reaction with DARPin-WT did not show fluorescence in the SDS-PAGE analysis, indicating that the fluorescence resulted from specific conjugation with an azide group incorporated into the target protein and does not occur with abortive synthesis products. This analysis showed that specific conjugation had occurred and that approximately 90% of the target protein with pAzF was conjugated.

The DARPin conjugate with the Cy5 fluorescent label, attached to the protein in the SPAAC reaction via pAzF and DBCO, was successfully used to stain mouse T-cells. In a sample in which cells were stained simultaneously with commercial CD3 and CD4 antibodies and anti-CD8 DARPin, the CD8^+^ T-cell population was strongly stained in the Cy5 channel and was clearly separated from the CD4 cell population (Figure 2C and Supplementary Figure S2 for FMO controls). This analysis confirms not only the conjugation of DBCO-Cy5 with the protein, but also that DBCO, attached to the C-terminus of the protein via AzF, does not prevent DARPin from effectively binding to its CD8 receptor epitope, which opens up opportunities for functionalizing the surface of LNPs while preserving the affinity properties of the DARPin protein.

After testing the conjugation technique with a fluorescent label, the conjugation with the lipid DSPE-PEG(2000)-DBCO was performed, followed by incorporation of the conjugate (IPAD-1) into lipid nanoparticles.

### 2.3. Preparation and Characterization of Lipid Nanoparticles Decorated with DARPin

**Figure 3:**
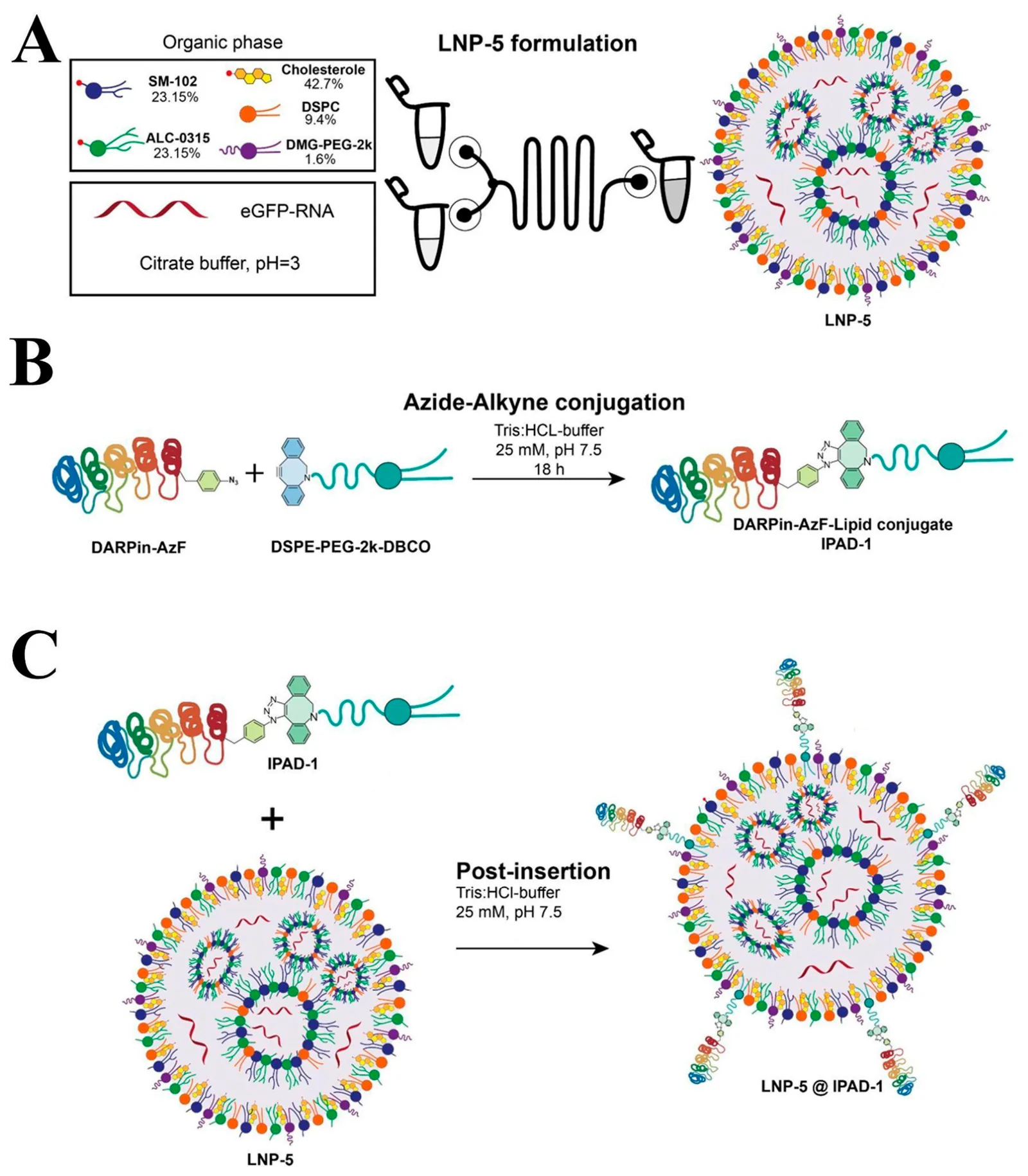
Design of formulations and decoration of LNP-5 by post-insertion method using conjugate DARPin-AzF with DSPE-PEG-2000-DBCO lipid (IPAD-1). (**A**) Schematic representation of the production of LNP-5. (**B**) Schematic representation of the production of the IPAD-1 lipid conjugate with DARPin-AzF using SPAAC. (**C**) Schematic representation of the production of decorated DARPin-LNPs by post-insertion of the IPAD-1 lipid conjugate into the formed particles.

To encapsulate mRNA-eGFP and deliver it to CD8^+^ T lymphocytes, we used microfluidic mixing of a lipid phase with an aqueous phase containing the mRNA. The LNP formulation consisted of two ionizable lipids (SM-102 and ALC-0315 in an equimolar ratio), cholesterol, DSPC, and DMG-PEG-2000 in a molar ratio of 23.15:23.15:42.7:9.4:1.6, respectively (Figure 3A), a composition previously used successfully for mRNA delivery [36]. The resulting LNP-5 platform was then used for further functionalization by the post-insertion method using the IPAD-1 conjugate (Figure 3C).

According to the latest literature, the post-insertion method is considered the most facile, as it does not require additional LNP formulations with a functionally anchored pegylated lipid via pre-mixing [20,37]. Preliminary tests on LNP formation using DSPE-PEG-2000-DBCO as a co-PEGylated component at different percentages relative to other lipids (0.27–1.60 mol%) yielded particles with a large PDI (>0.2) and Z-average (>200 nm) after dialysis against PBS (pH 7.4). The situation improved significantly after replacing PBS with Tris-HCl buffer at the same pH. However, subsequent direct decoration with DARPin-AzF and isolation of decorated LNP-5 resulted in complete loss of encapsulated contents during purification by tangential flow filtration. In contrast, the post-insertion method for IPAD-1 produced colloidally stable materials that were suitable for in vitro testing. The molar ratio of IPAD-1 relative to the total lipid mixture in LNP-5 was chosen in the range of 0.10–0.27 mol% by analogy with the data of O.Heidenreich and co-authors on the post-insertion of DSPE-PEG-2000-DBCO conjugated with peptide azido-LDV or dye azido-Cy3 [38].

All LNP-5 formulations, including those targeted to CD8^+^ cells, had to meet the generally accepted quality control criteria for LNP–mRNA materials: Z-average (80–120 nm), PDI (0.05–0.20), and encapsulation efficiency (EE, >85%). To compare physicochemical properties, we prepared samples containing different percentages of IPAD-1 (0.10 and 0.27 mol%, respectively) and isolated them by tangential flow filtration in different buffers (PBS and Tris-HCl).

**Figure 4:**
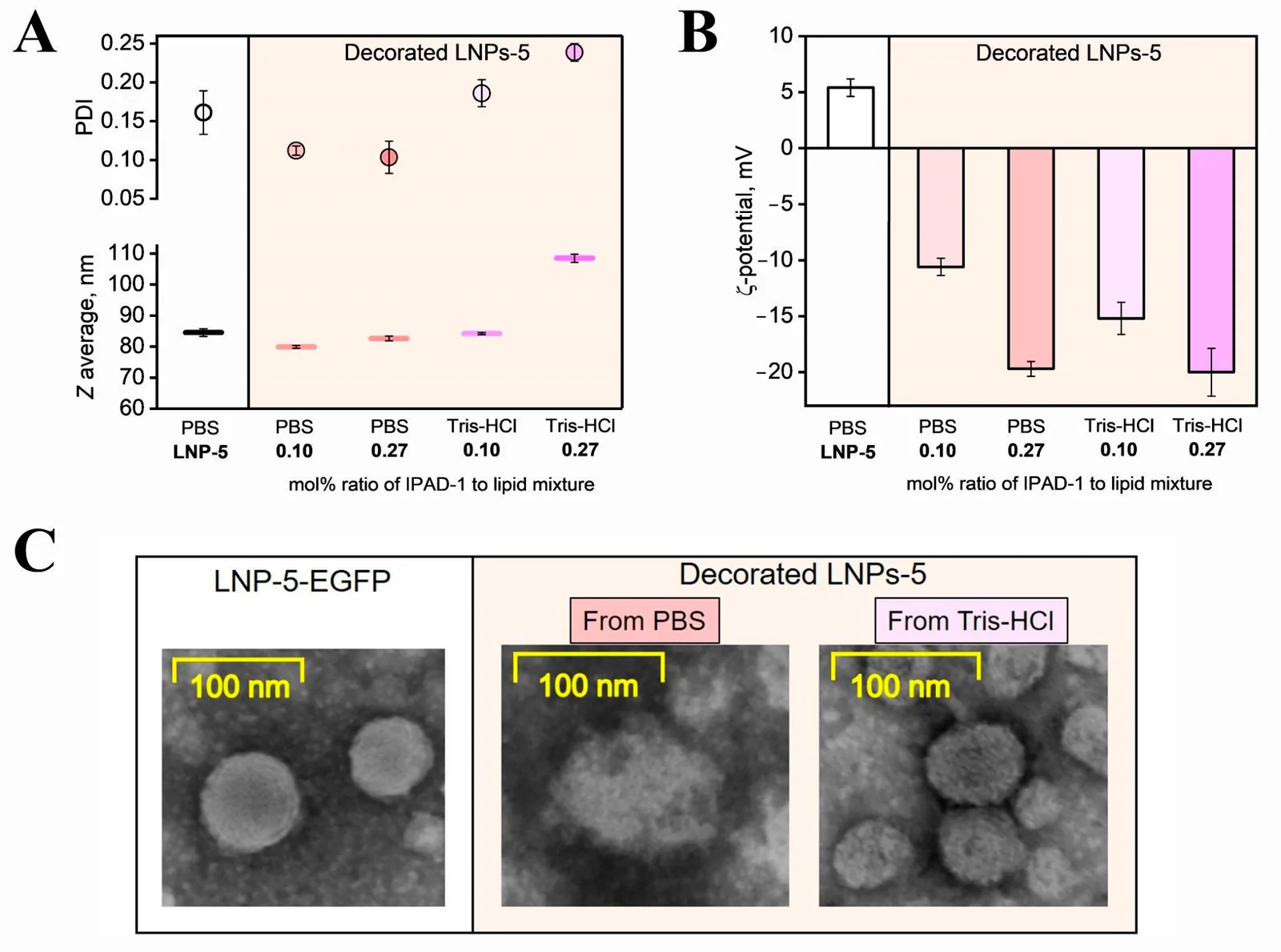
Comparison of ζ-potential (**A**), polydispersity index (**B**) and average hydrodynamic diameter (Z average, (**B**)) depends on buffer nature for initial LNP and LNPs after decoration by post-insertion of IPAD-1 with different IPAD-1-to-PEG molar ratios (0.10:1.5 and 0.27:1.33 mol%). (**C**) Micrographs for initial LNP-5—white box, after decoration—peach box. On the left, in a peach box, a micrograph of decorated LNPs dialyzed against PBS (pH 7.4) is shown; on the right—against Tris-HCl (pH 7.4).

The main distinguishing feature between unmodified LNP-5 and decorated LNP-5@IPAD-1 was the change in ζ-potential (Figure 4A). The initial LNP-5 showed a slightly positive ζ-potential (+5.5 mV), whereas the decorated particles, regardless of buffer type or IPAD-1 molar content, exhibited a reversal of the surface charge (a shift to −10 to −20 mV), likely due to the combined contribution of the negative ζ-potential of DARPin and the phosphate groups of DSPE-PEG-2000. This may indicate incorporation of IPAD-1 into the outer lipid layer.

In almost all cases, post-insertion did not increase the average hydrodynamic diameter, with the exception of particles isolated from PBS and containing 0.27 mol% IPAD-1. In this case, an increase in the concentration of the protein–lipid conjugate in LNP-5 raised the Z-average to 108 nm and the PDI to 0.24 (Figure 4B), indicating a disruption of the spherical structure which led to nanoparticle aggregation and release of the encapsulated mRNA in subsequent experiments on cell cultures.

STEM micrographs with negative uranyl acetate staining enabled visualization of the structure and morphology of the nanosystems. Control LNP-5 nanoparticles had a regular spherical shape with well-defined boundaries, as previously observed for similar LNP-5–based systems [36]. However, when nanoparticles were modified and isolated from PBS, their morphology changed: predominantly shapeless fragments lacking the original spherical form were observed (Figure 4C). Such structural disruption in PBS may indicate that electrostatic interactions with IPAD-1 in this medium destabilize the lipid bilayer or induce a phase transition leading to LNP-5 disintegration. Nanoparticles obtained in Tris-HCl buffer retained a more pronounced rounded structure; their surface appeared rough, loose, and covered with a dense molecular layer. Successful incorporation of the DARPin–lipid conjugate was evidenced by the reversal of ζ-potential and by STEM morphology. However, the exact number of DARPin molecules per particle was not determined in this study.

Table 1 summarizes all the obtained physicochemical characteristics of the nanosystems. All LNPs exhibit a high degree of encapsulation before and after post-insertion (EE 90–100%). Notably, despite the high EE (97%) observed for 0.27 mol% IPAD-1 content and the use of PBS as a buffer for modification and isolation of decorated particles, the total particle concentration per unit volume measured by the MADLS method decreased twofold relative to the initial LNP-5. In contrast, all types of particles isolated using Tris-HCl remained at approximately 3.5 × 10^12^ particles/mL.

Thus, through the formation, post-insertion decoration, and systematic physicochemical study of LNPs, we determined the optimal buffer and IPAD-1-to-PEG ratio for obtaining optimal lipid nanoparticles for subsequent cytofluorimetric experiments. Although both 0.10 and 0.27 mol% IPAD-1 could be incorporated, the 0.10 mol% formulation in Tris-HCl buffer showed superior colloidal properties (Z-average ≈ 83 nm, PDI ~ 0.10) and was used for the functional studies. This optimized formulation (0.10 mol% IPAD-1 in Tris-HCl) was used for all subsequent transfection studies. Physicochemical properties of the maleimide-conjugated control LNPs were comparable to those of the IPAD-1 decorated particles and are summarized in Supplementary Table S2.

### 2.4. Click Chemistry Provides CD8^+^ Targeting Efficiency Comparable to Maleimide-Based Conjugation

**Figure 5:**
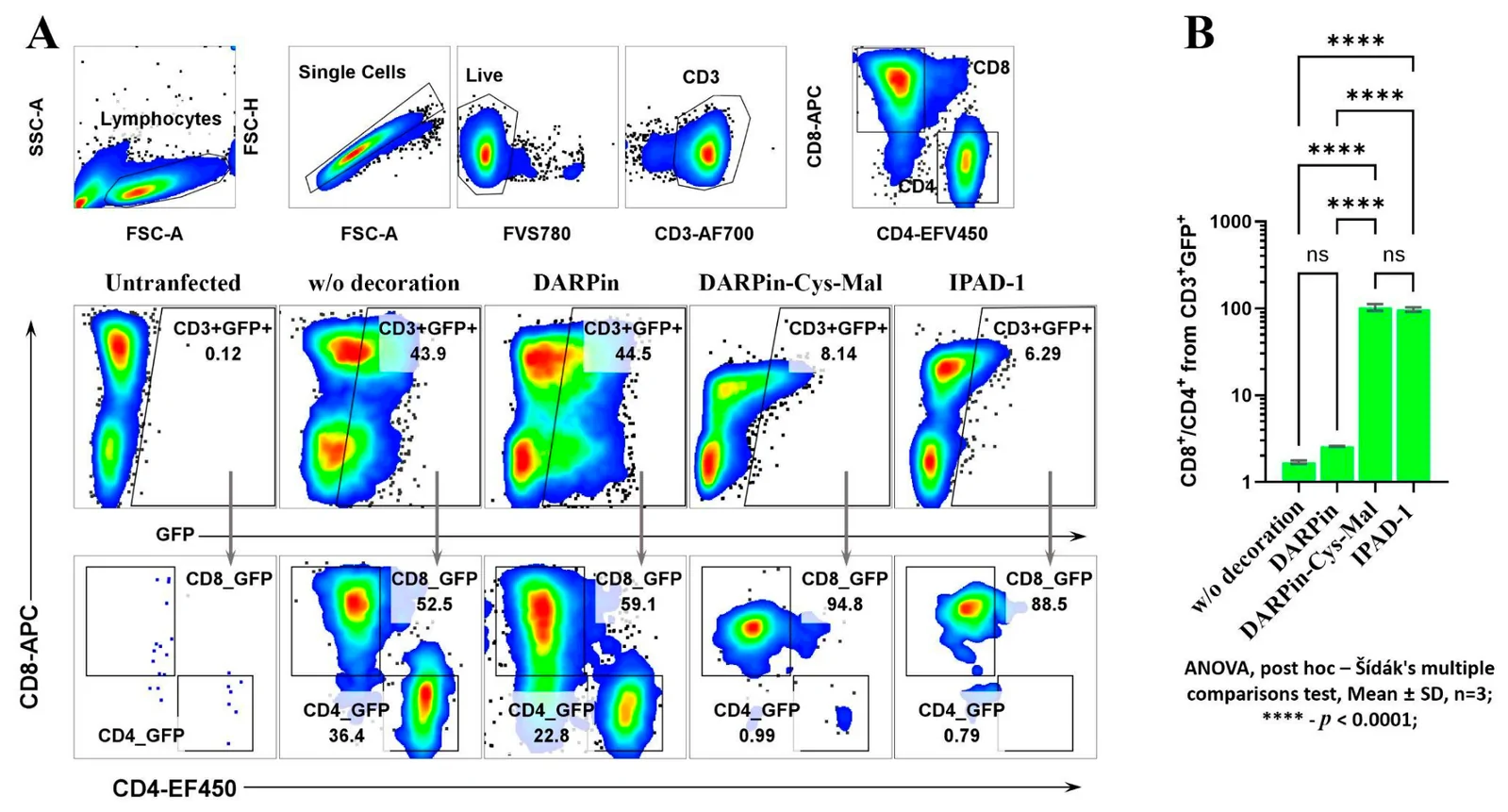
Specificity of CD8-targeted LNP decorated with DARPin via click chemistry or maleimide conjugation. (**A**) Gating strategy and representative dot plots showing GFP expression in CD3 T-cells and distribution between CD8^+^ and CD4^+^ subsets after treatment. (**B**) CD8/CD4 ratio among CD3^+^GFP^+^ lymphocytes. Data presented as Mean ± SD. **** *p* < 0.0001, ns—not significant; One-way ANOVA, post hoc—Šidák multiple comparison test.

The specificity of targeting was evaluated in vitro by assessing the selective transfection of murine CD8^+^ T lymphocytes with eGFP mRNA–LNPs decorated with anti-CD8 DARPin-pAzF (IPAD-1 group). All functional experiments were performed with the optimal formulation identified in the physicochemical characterization (0.10 mol% IPAD-1, post-insertion and isolation in Tris-HCl buffer). For comparison, we used undecorated LNPs, LNPs with passively adsorbed DARPin-AzF (DARPin group), and LNPs decorated with a reference anti-CD8 DARPin conjugated via a maleimide–thiol bond to DMG-PEG-2000-Mal (DARPin-Cys-Mal). Untransfected cells served as a negative control (Figure 5A).

Analysis of GFP expression in CD4^+^ and CD8^+^ cells showed that undecorated LNPs, as well as LNPs with passive decoration, non-specifically transfect both lymphocyte populations. In contrast, targeted conjugation of both anti-CD8 DARPin variants led to a sharp increase in the proportion of GFP-positive events in the target population of CD8^+^ lymphocytes and, at the same time, reduced nonspecific transfection of CD4^+^ lymphocytes to background levels. Blocking the CD8 receptor on T cells with a commercial antibody (clone 53-6.7) before adding the particles decorated with IPAD-1 reduced transfection by about 20-fold to almost zero (Supplementary Figure S3).

To evaluate targeting specificity, we calculated the ratio of CD8^+^ cells to CD4^+^ cells among GFP-positive T lymphocytes. As expected, for undecorated LNPs this value was minimal (1.7 ± 0.07), reflecting the lack of specificity. Passive decoration with DARPin particles containing pAzF did not result in specific transfection of CD8^+^ lymphocytes, as evidenced by the distribution of CD4+GFP+ and CD8+GFP+ cells, which remained comparable to that observed in the group transfected with unmodified PEG particles. This finding highlights that achieving specificity of decorated particles requires the use of a protein–PEG conjugate, and not just protein alone, since passive protein adsorption is insufficient to ensure target selectivity. At the same time, both variants of directed conjugation of LNPs with the CD8 receptor led to a significant increase in the selectivity index to ~90 (Figure 5B). Thus, the efficiency and specificity of LNP targeting using bioorthogonal click chemistry are comparable to those achieved with the classical maleimide conjugation method.

## 3. Discussion

In this pilot study, we developed and validated a novel DARPin-LNP-based platform for targeted mRNA delivery to mouse CD8^+^ T-cells. Our approach combines several key innovations: biosynthetic production of DARPin with pAzF modification through genetic code expansion and a novel strategy for post-insertion of a protein–lipid conjugate into LNPs. The introduction of a single pAzF functional group at a specific position in the protein enabled copper-free click chemistry using DBCO for functionalization of the LNP surface.

**Figure 6:**
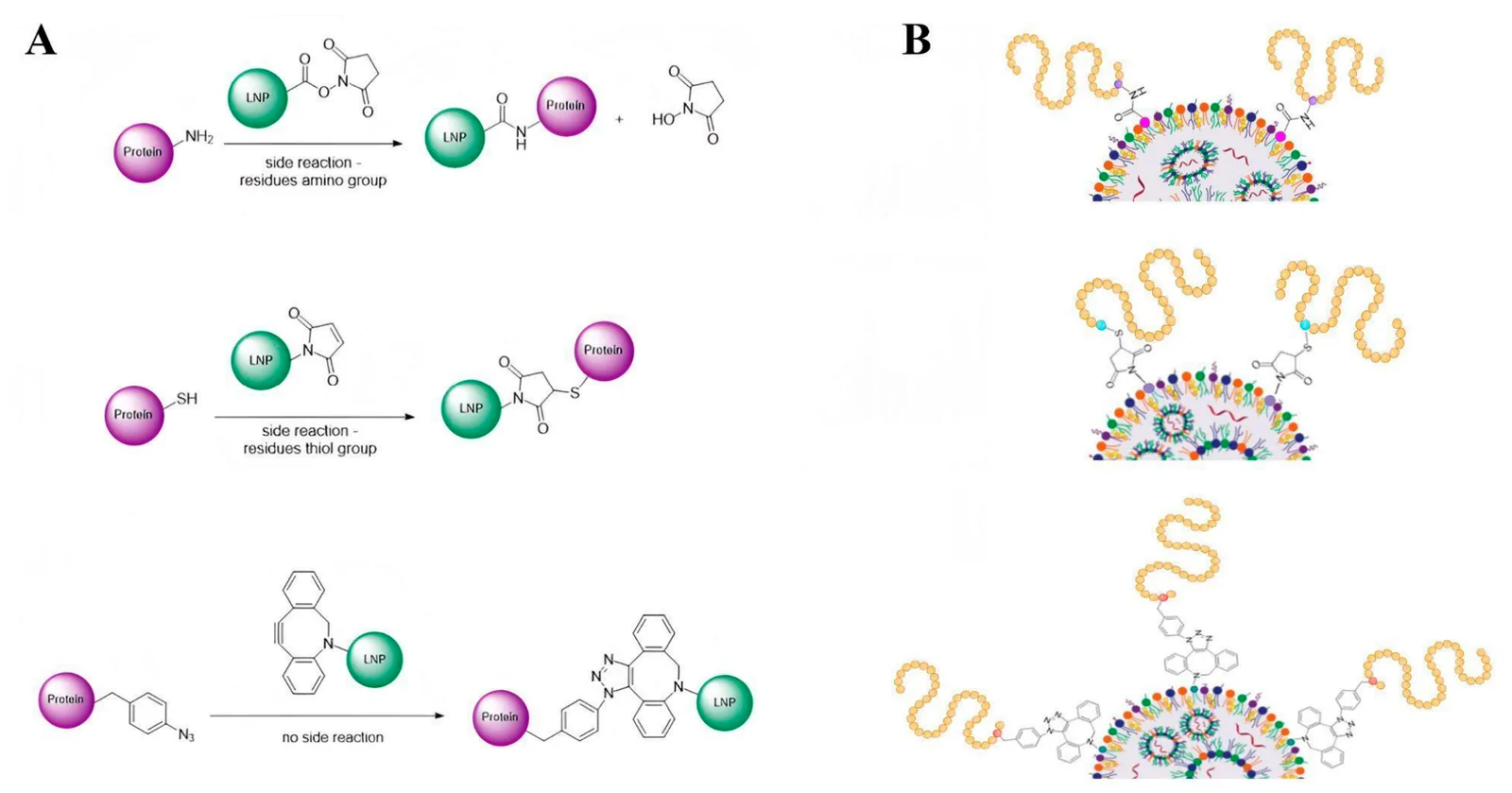
(**A**) Various conjugation methods for attaching proteins to LNPs. (**B**) Schematic representation of the protein on the LNP surface under different types of conjugation.

Traditional approaches to covalently cross-link proteins to the lipid components of LNPs rely on classical carbodiimide coupling reactions, amide coupling via activated esters, or thiol–maleimide chemistry (Figure 6) [39].

Modification of lysine residues using lipids activated with N-hydroxysuccinimide esters or direct cross-linking of carboxylated lipids to the protein using EDC/NHS is the most accessible method [40]. However, according to the literature, this method has several drawbacks. Because of the large number of accessible amino groups on the protein surface, the reaction proceeds stochastically, resulting in a heterogeneous mixture of proteins with varying degrees of modification. Unpredictable protein binding also leads to incorrect spatial orientation on the LNP surface [41]. In addition, heterogeneity of the polydispersity index of lipid nanoparticles is observed, since uncontrolled protein cross-linking between several nanoparticles or protein multimerization on a single particle disrupts the structural stability of the lipid layer. Disruption of uniform lipid packing leads to local defects, induces aggregation, increases nanoparticle size, and causes irreproducibility of the protein/nanoparticle ratio from batch to batch [42]. Moreover, the use of EDC/NHS activation for nanoparticle functionalization with proteins is associated with kinetic and structural limitations, since conjugation efficiency critically depends on pH, buffer ionic strength, and control of side reactions [43,44].

To achieve greater selectivity, the Michael reaction between maleimide-functionalized lipids (DMG-PEG-2000-Mal) and free sulfhydryl groups of protein cysteines is often used [45]. Although this reaction occurs under relatively mild conditions and is rapid, its adaptation for LNP modification is not always feasible. Toxicology studies have demonstrated that residual unreacted maleimide groups on the surface of lipid nanoparticles cause pronounced immunogenicity and activation of the complement system [46]. Free maleimide on the surface of nanostructures has been shown to actively bind serum albumin in vivo. Albumin clustering on the LNP surface triggers the alternative pathway of the complement cascade, leading to accelerated particle clearance from the bloodstream, a 50% decrease in platelet levels, and severe systemic toxicity. Another problem is the chemical instability of the conjugate: the succinimide-thioester bond undergoes a reversible process in vivo [47]. Retro-Michael opening (retro-Michael reaction) can occur. In the presence of high concentrations of endogenous thiols, such as glutathione or free plasma cysteines, the targeting protein is gradually cleaved from the LNP surface, which can lead to premature release of the encapsulated cargo [48]. In addition, the maleimide ring undergoes spontaneous hydrolysis in aqueous solutions. On the one hand, the opened ring stabilizes the conjugate and stops the retro-reaction. This process occurs stochastically, creating chemical heterogeneity (a mixture of closed and opened forms), which complicates standardization [49]. While the SPAAC linkage used in the present work is expected to be more stable than the maleimide–thiol bond, serum stability and long-term storage data for the DARPin–lipid conjugate were not evaluated in this study and will be important to confirm in future work.

An oriented insertion implies that the protein is attached to the lipid nanoparticle at a strictly defined site, leaving the active site exposed. This is difficult to achieve with thiol–maleimide chemistry. First, if the protein has several free cysteines, the maleimide will bind to all of them randomly. The structure will then be fixed in chaotic spatial orientations, partially shielding the protein’s active site [13]. However, this can be avoided by selecting proteins or peptides that lack cysteines, for example, DARPin, as the targeting agent. Second, to achieve oriented insertion, a cysteine tag is often artificially introduced into the C- or N-terminus of the protein. However, free thiol groups frequently lead to unwanted dimerization or aggregation of molecules during production or purification [50]. To carry out conjugation, incorrectly bound cysteines must be released using reducing agents such as DTT or TCEP [51]. In complex proteins and full-length antibodies, disulfide bond formation is critical for establishing tertiary structures and interdomain links. Attempts to partially restore these bonds for targeted conjugation can lead to complete denaturation, structural disruption, and loss of biomolecular functionality.

These limitations of classical conjugation methods motivated the use of a bioorthogonal, site-specific strategy in the present work. Protein conjugates with targeted modification have repeatedly demonstrated pharmacological profiles that are generally superior to those of their heterogeneous counterparts. Among existing methods for targeted protein modification, incorporation of ncAAs via genetic code expansion is a cutting-edge approach, as stoichiometry and conjugation site can be precisely controlled using bioorthogonal chemistry. The application of this method has recently gained widespread attention due to clinical trials (NCT04829604 and NCT04009681) of therapeutics based on proteins containing ncAA.

The approach proposed in our study, based on pAzF and click chemistry, helps to overcome the previously described limitations of protein conjugation to LNPs. The platform relies on the site-specific introduction of pAzF into the DARPin protein, which targets the mCD8 receptor. We developed an efficient protocol for producing DARPin containing functional pAzF at the C-terminus of the sequence. Recombinant DARPin–pAzF was produced using an orthogonal tRNA/aminoacyl-tRNA synthetase pair in an *E. coli* expression system directly during biosynthesis in producer cells, completely eliminating complex and expensive in vitro chemical modifications. This ensures reproducible production and makes the method scalable.

Another key advantage of the developed protocol for producing DARPin with ncAA is the cleavage of affinity tags using enteropeptidase. Removal of affinity tags before LNP decoration is necessary to eliminate their antigenicity, which can induce T- and B-cell immune responses and reduce the therapeutic index of the drug upon repeated administration. Furthermore, elimination of the His-tag removes steric hindrances that compromise protein conformational stability.

For subsequent decoration of lipid nanoparticles, preconjugation was performed via a click chemistry (SPAAC) reaction between the azide group of DARPin-pAzF and the dibenzocyclooctyne moiety of DSPE-PEG-2000-DBCO lipid. SPAAC reactions occur under mild physiological conditions at neutral pH and room temperature, which helps to preserve the native protein structure. In this study, we investigated an approach using copper-free click chemistry combined with a post-insertion step into preformed LNPs after dialysis. We found that this method effectively incorporates DSPE-PEG-2000-DBCO without significantly affecting the physicochemical parameters or encapsulated content of preformed LNPs, compared to the classical direct decoration method. Direct incorporation of DBCO lipids at varying ratios negatively affected the physicochemical properties of the resulting particles, and subsequent decoration, according to our data, resulted in complete loss of the encapsulated content. Additionally, we found that post-insertion is more efficient in more stable buffer systems, such as 25 mM Tris (pH 7.5), yielding particles with a favorable polydispersity index and size compared to those in PBS. PBS leads to particle deformation and the generation of shapeless fragments during LNP-5 decoration. Thus, the choice of buffer critically affects the structural integrity of the modified systems. Tris-HCl helps preserve nanoparticle integrity while promoting the formation of a developed porous surface layer.

The resulting protein-LNP conjugates showed effective targeting of the CD8^+^ T-cell population isolated from mouse splenocytes. Flow cytometry confirmed that the targeting efficiency of our LNPs is significantly higher, with markedly increased uptake by CD8 cells and minimal off-target effects on non-CD8-expressing cells. Recently, Buchholz and colleagues developed a platform for decorating LNPs with the fusion protein ApoE2–DARPin, using the same MSE10 DARPin clone used in our work [16]. Following transfection of activated murine splenocytes, they obtained a similar outcome: approximately 7.5% of CD3^+^ cells were transfected, and all of these were CD8^+^ cells. These findings are in good agreement with our data, both in terms of overall transfection efficiency and selectivity for the CD8^+^ target population.

However, a discrepancy arises when comparing nondecorated LNPs. In the Buchholz study, particles resulted in negligible transfection of murine T cells (<1%), whereas in our study the same condition yielded 43.9% eGFP-positive cells among the total CD3^+^ population. This marked difference is likely attributable to variations in the lipid composition of the two formulations. While their LNPs contained the ionizable lipid Dlin-MC3-DMA, our particles were formulated with an equimolar combination of SM-102 and ALC-0315. As a result, the apparent targeting advantage of the DARPin-decorated particles in our study is measured against a relatively high baseline transfection of the undecorated formulation. This hypothesis is supported by the work of Escalona-Rayo et al., who demonstrated in murine bone-marrow-derived dendritic cells (BMDCs) that both cellular uptake and eGFP expression were influenced by the type of ionizable lipid, with the efficiency ranking SM-102 > ALC-0315 > Dlin-MC3-DMA [52].

These observations underscore that the transfection outcome of targeted LNP systems is governed by a complex interplay of multiple parameters. The choice of ionizable lipid is a critical determinant of intrinsic delivery potency, yet it does not suffice to ensure cell-type specificity. Equally important is the method of decoration: passive adsorption of targeting proteins fails to confer selectivity, whereas covalent conjugation through well-defined chemistry provides stoichiometric, oriented display of the ligand on the LNP surface. In our study, the click reaction between DBCO and the pAzF residue in the DARPin successfully achieves this stable, stoichiometric, oriented configuration.

Some parameters, in particular the absolute surface density of ligands, broader dose–response profiles, and kinetic changes beyond the 24 h interval, have yet to be quantified. Although the consistent shift in ζ-potential and the change in particle morphology after post-insertion confirm successful incorporation of the DARPin–lipid conjugate, precise surface density of DARPin would further strengthen claims of batch-to-batch reproducibility and facilitate direct comparison with other conjugation strategies. Furthermore, this initial validation of the click-chemical LNP conjugation platform was restricted to in vitro models, leaving in vivo animal evaluations for subsequent stages. Rather than reflecting limitations of the methodology itself, the omission of these parameters reflects the focused scope of this study, which was to create and verify the platform for LNP functionalization based on click chemistry using a biotechnological approach to embedding azido-L-phenylalanine into a targeted protein. We used DARPin against mouse CD8 as a model ligand to demonstrate the fundamental feasibility of this approach. In subsequent work, we plan to systematically quantify ligand stoichiometry, construct complete dose–response and kinetic transfection curves, replace mouse DARPin with a human equivalent for validation on primary human cells, and move to in vivo assessments to advance this platform for use in mRNA vaccines and in vivo CAR-T cell engineering.

Thus, our approach, which relies on site-specific incorporation of pAzF into the amino acid sequence of the target protein followed by bioorthogonal click conjugation on the LNP surface, represents a universal platform technology. The proposed strategy is not limited to DARPin but is applicable to a wide range of recombinant proteins and peptides. Due to the high selectivity and mild reaction conditions of the azide-alkyne cycloaddition, this method enables controlled, spatially directed functionalization of nanocarriers, opening opportunities for flexible surface decoration of LNPs. This versatility allows the platform to be adapted for diverse therapeutic modalities beyond immunotherapy, including targeted delivery of mRNA vaccines, CRISPR-Cas9 gene editing components, and cell reprogramming factors, thus providing a unified chemical toolkit for precision delivery across multiple biomedical applications.

## 4. Materials and Methods

### 4.1. Plasmids and Molecular Cloning

The following working concentrations of antibiotics (Gold Biotechnology, St. Louis, MO, USA) were used to prepare selective media: carbenicillin, 50 μg/mL; spectinomycin, 50 μg/mL; chloramphenicol, 30 μg/mL; kanamycin, 30 μg/mL; tetracycline, 15 μg/mL; streptomycin, 50 μg/mL. p-Azido-L-phenylalanine (Tokyo Chemical Industry Co., Ltd., Tokyo, Japan) was used in this study.

All oligonucleotides were synthesized at the Scientific Research Center of the Institute of Geology and Microbiology of the National Technical University “Sirius” using an ASM-800ETf DNA/RNA synthesizer (BIOSSET, Novosibirsk, Russia).

The sequence encoding the catalytically active light chain of human enteropeptidase (hEKL) was obtained from publicly available databases [33] and assembled by PCR from overlapping oligonucleotides using flanking primers. The hEKL gene was cloned into the pET30(a+)_His8_Trx_Enterokinase plasmid. The pET30(a+) plasmid (Addgene #175021) was used as the vector.

Plasmids containing the mouse anti-CD8 DARPin genetic sequence were assembled in a similar manner.

Assembly and subsequent steps for plasmid DNA production and purification were performed in the same way for all constructs. PCR was performed using Q5 Hot Start High-Fidelity DNA Polymerase (New England Biolabs, Ipswich, MA, USA). Plasmids were produced using isothermal assembly with Gibson Assembly 2x Master Mix (New England Biolabs). E. cloni^®^ 10G (LGC Biosearch Technologies, Teddington, UK) was used for nucleic acid production. Cells were seeded on Petri dishes containing LB agar and the appropriate antibiotic. The following day, 10–20 colonies were screened, followed by overnight culture and plasmid DNA isolation using the Plasmid Miniprep Kit (Biolabmix, Novosibirsk, Russia). Sequence verification was performed by Sanger sequencing on an Applied Biosystems 3730xl DNA Analyzer (48 capillaries, 50 cm) (Thermo Scientific™, Massachusetts, MA, USA) using POP-7 Polymer.

All plasmid vectors used in this study are listed in Supplementary Table S1.

### 4.2. Production and Purification of Proteins

SHuffle^®^ T7 Express electrocompetent cells (New England Biolabs, USA) were co-transformed with pET30a(+)_His8_Trx_hEKL_Enterokinase and pGro7 (Addgene #228495). After colonies were obtained on a Petri dish, an overnight culture was inoculated into LB medium supplemented with kanamycin and chloramphenicol and grown at 37 °C, 180 rpm overnight. For 1 L of liquid LB medium, the following were added: appropriate antibiotics, arabinose to a final concentration of 10 µM, and overnight culture until an optical density of OD_600_ = 0.1 was reached, measured using a NanoDrop (Thermo Fisher Scientific, Waltham, MA, USA). Cells were cultured at 30 °C until an optical density of OD_600_ = 1.2 was reached, and IPTG was added to a final concentration of 0.7 mM. After induction of protein expression, cells were grown in a shaker incubator at 25 °C for 24–30 h.

Electrocompetent T7 Express cells (New England Biolabs, USA) were co-transformed with two plasmids: pET30(a+)_His8_DDDDK_CD8_DARPin_ncAA, containing an amber stop codon, and the pHPyl-AzFPylRS-GlnS plasmid containing an orthogonal APC that incorporates pAzF during translation. Transformants were plated on media containing kanamycin or kanamycin and chloramphenicol, respectively, and incubated at 37 °C for 16–18 h. After colony formation, individual clones were screened to confirm the presence of the correct plasmid. The best clones were inoculated into 7 mL of liquid LB medium and incubated at 37 °C, 180 rpm for 16–18 h. The overnight culture was then added to a flask containing 500 mL of 2xYT broth-based induction medium to an OD_600_ = 0.1, supplemented with appropriate antibiotics and, where required, pAzF to a final concentration of 1 mM. Cells were grown to OD_600_ = 1.2 at 37 °C, 180 rpm. When the required density was reached, 1 mM IPTG was added, and the culture was incubated for 24 h at 37 °C, 180 rpm.

After expression, the cell suspension was centrifuged in an Eppendorf Centrifuge 5910R at 6000 rpm for 15–20 min at 4 °C. The supernatant was discarded, and the cell pellet was resuspended in 30 mL of buffer (pH 7.8, 50 mM Tris, 0.005 mM imidazole, 0.3 M NaCl) or frozen at −20 °C until required. Cell lysis, IMAC chromatography, and mass spectrometric analysis of samples were performed as previously described [34].

Purified proteins were dialyzed in a 10 kDa cellulose bag (Thermo Fisher Scientific, USA) against storage buffer (pH 7.5, 25 mM Tris, 2 mM CaCl_2_). The resulting enterokinase was diluted to a concentration of 0.1 mg/mL. The resulting mCD8 DARPin was diluted to a concentration of 1 mg/mL. Enteropeptidase was then added to DARPin at one tenth of the volume. The resulting protein mixture was incubated at 20 °C for 12–16 h.

After completion of enzymatic digestion, further purification of mCD8 DARPin was performed by anion-exchange chromatography using Smart Sep Q30 sorbent on a Bio-Rad chromatography system (Bio-Rad, Hercules, CA, USA). Before sample application, the sorbent was equilibrated with buffer containing 25 mM Tris-HCl (pH 7.5). The protein mixture was applied to the column and then washed with equilibration buffer until unbound components were removed. Elution was carried out with a linear gradient of elution buffer (25 mM Tris-HCl, 1 M NaCl, pH 7.5) from 0 to 100% over 60 mL at a flow rate of 1 mL/min. The resulting fractions were analyzed by SDS-PAGE.

### 4.3. Protein Electrophoretic Separation in PAGE

To visualize the protein obtained after chromatographic purification, SDS-PAGE was performed using a vertical electrophoresis system from Bio-Rad, USA. 2–10 micrograms of protein were heated to 95 °C for 10 min in 4× Laemmli Sample Buffer (Bio-Rad, USA) with 5% β-mercaptoethanol, and then separated using a 16% separating gel and a 5.0% concentrating gel. A marker, RAV-11 (Biolabmix, Russia), in a volume of 3 µL was loaded into an empty well. The protein bands were stained with Coomassie Brilliant Blue Ultrafast Staining Solution (Servicebio, Wuhan, China) in accordance with the manufacturer’s instructions. After electrophoresis, fluorescent images were obtained at 630 nm, as well as a colorimetric image of the Coomassie-stained gel, using the Bio-Rad ChemiDoc Imaging System (Bio-Rad, USA) gel documentation system.

### 4.4. Click Chemistry Conjugation

DARPin MSE10 was diluted to a concentration of 1 mg/mL in buffer (25 mM Tris, pH 7.5). DBCO-Cy5 (Cat. no. 30F0, purchased from Lumiprobe, Moscow, Russia) was diluted to 1 mg/mL in DMSO. DSPE-PEG(2000)-DBCO (1,2-distearoyl-sn-glycero-3-phosphoethanolamine-N-[dibenzocyclooctyl(polyethylene glycol)-2000]) (Avanti, Birmingham, AL, USA) was diluted to a concentration of 10 mg/mL in DMSO. The protein solution was mixed with excess fluorescent label or lipid at a molar ratio of 1:5 to ensure complete binding. The solution was incubated for 16–18 h at 4 °C. Upon completion, unreacted compounds were removed using a 10 kDa cutoff centricon (Millipore, Darmstadt, Germany). The conjugates were stored at 4 °C for short-term use and at −80 °C in aliquots for long-term use. Concentrations were determined using the QuDye^®^ Protein Assay Kit (Lumiprobe, Russia).

### 4.5. In Vitro Transcription and RNA Purification

The DNA construct encoding eGFP mRNA used in this work was prepared using the commercial pSmart vector (Lucigen, Middleton, WI, USA), which was linearized by the endonuclease AhlI (SibEnzyme, Moscow, Russia) for in vitro transcription. The mRNA structure included CapAG, the 245-nucleotide viral 5′ UTR of TPL, the eGFP coding sequence, the 3′ UTR from the mRNA vaccine mRNA-1273 (Moderna, Cambridge, MA, USA), and a 114-nucleotide poly(A) tail. mRNA synthesis was performed in IVT buffer from the mRNA-20 in vitro mRNA synthesis kit (Biolabmix, Russia). The reaction mixture also included 10 mM DTT (Biolabmix, Russia), 1 U/μL RiboCare RNase inhibitor (Evrogen, Moscow, Russia), 18 U/μL T7 RNA polymerase (Biolabmix, Russia), 500 ng linearized template DNA, 2.4 mM cap analog AG (Biolabmix, Russia), and 3 mM each of the four ribonucleoside triphosphates (Biolabmix, Russia). m1ΨTP was used instead of UTP to reduce RNA immunogenicity. The final volume of the reaction mixture was 50 μL. Reactions were incubated for 2 h at 37 °C. Template DNA was digested with thermolabile DNase (2 U, 15 min, 37 °C). mRNA was purified using VAHTS RNA Clean Beads (Vazyme, Nanjing, China), and quality was assessed by capillary gel electrophoresis on Qsep (Bioptic, Changzhou, China; Supplementary Figure S1).

### 4.6. LNP Formulation

To encapsulate mRNA-eGFP into lipid nanoparticles, we used the Automated NP System for liposome generation (micromixer chip, Dolomite Microfluidics, Royston, UK). The formulation technique was similar to that described for other mRNA platforms [53]. The aqueous phase was 10 mM citrate buffer at pH 3 (RusChem, Moscow, Russia); the organic phase was absolute ethanol (Hayman, Mumbai, India). The RNA concentration was 200 ng/μL in citrate buffer (pH 3). The total lipid concentration in ethanol was 10 μg/μL. The lipid composition included cholesterol (Merck, Darmstadt, Germany), ICL SM-102 (Sinopeg, Xiamen, China), ICL ALC-0315 (Sinopeg, China), helper lipid DSPC (Avanti, USA), and pegylated lipid DMG-PEG2000 (Avanti, USA). The optimal molar ratio of the components was 42.70:23.15:23.15:9.40:1.60 mol% (cholesterol:SM-102:ALC-0315:DSPC:DMG-PEG2000). The volume ratio of the aqueous phase to the lipid phase was 3:1, and the total flow rate was 1.5 mL/min. After formulation, particle suspensions were purified by dialysis using 1 mL Float-A-Lyzer G2 membranes with a molecular weight cutoff (MWCO) of 10 kDa; the membrane material was Cellulose Ester (Repligen, Waltham, MA, USA). Membranes were prepared according to the manufacturer’s protocol. Dialysis was performed overnight (12 h) in phosphate-buffered saline (PBS, pH 7.4) at +4 °C with vigorous stirring. The buffer was changed once. For LNPs containing DMG-PEG(2000) at molar ratios of 1.33% or 1.5%, DSPE-PEG(2000)-DBCO DARPin conjugate was added to achieve molar ratios of 0.27% or 0.1%, respectively. For this study, DARPin-AzF nanoparticles relying on passive adsorption were generated as a reference group. To immobilize the target proteins on the LNP surface, equimolar amounts of protein (lacking preconjugated DSPE-PEG-DBCO) were incubated for 60 min. After 1 h incubation with gentle shaking at room temperature, LNPs were dialyzed against 25 mM Tris buffer (pH 7.5) using centrifugal concentrators with a 100 kDa mass cutoff (Jet Biofil, Guangzhou, China). Additionally, control LNPs decorated with anti-CD8 DARPins—covalently attached via a maleimide-thiol interaction to the DMG-PEG-2000-Mal lipid anchor—were included in the experiment (Supplementary Table S2).

### 4.7. Physicochemical Characteristics of LNPs

#### 4.7.1. Dynamic Light Scattering

LNP size and polydispersity index (PDI) were measured using a Zetasizer Ultra (Malvern Panalytical, Malvern, UK). LNPs were diluted 100-fold in PBS (pH 7.4) and equilibrated at room temperature before analysis in a plastic cuvette for particle size measurements. Three measurements of up to 100 runs were collected for each sample until the value stabilized.

#### 4.7.2. ZetaPotential

Zeta potential was measured using a Zetasizer Ultra (Malvern Panalytical) equipped with a U-type cuvette. LNPs were diluted 100-fold in deionized water and equilibrated at room temperature before analysis. Three measurements for three samples of up to 100 runs were collected for each sample until the value stabilized. The relative standard deviation did not exceed 5%.

#### 4.7.3. Encapsulation Efficiency

Encapsulation efficiency (EE, %) was evaluated using a previously described procedure [53]. To assess encapsulation, particles were lysed in TE buffer at pH 7.5 with 1% Triton X-100, and both lysed and non-lysed particles were stained with the intercalating dye SYBR Green reagent (Eurogene, Moscow, Russia) before and after LNP disruption. RNA content in the particles was quantified using a calibration curve of the mRNA standard in TE buffer, with and without Triton X-100. The mRNA concentration was determined by calculating the difference in detected mRNA between lysed and non-lysed particles.

#### 4.7.4. Scanning Transmission Electron Microscopy (STEM)

STEM micrographs of the lipid nanoparticles were obtained using a Crossbeam 550 scanning electron microscope (Carl Zeiss, Oberkochen, Germany) with a transmission electron microscopy detector (accelerating voltage 30 kV, current 200 pA). Before recording micrographs, freshly formulated LNPs (total lipid concentration 10 mg/mL) were diluted 10-fold with PBS, and 10 μL aliquots were collected for analysis. The resulting suspension (10 μL) was applied to a small piece of Parafilm. Next, a copper grid for STEM was transferred to a drop of the sample and incubated for 2 min. Then, 10 μL of contrast agent (1% aqueous uranyl acetate solution) was applied to the grid with the sample. Incubation was continued for an additional 1 min. The grid was dried at room temperature. STEM images were recorded immediately after the described sample preparation.

### 4.8. Isolation and Activation of Mouse Splenocytes

A female Balb/c mouse was euthanized at 8 weeks of age. Animal experiments were approved by the Ethics Committee of Sirius University of Science and Technology (N 6.1) on 15 January 2024. The spleen was mechanically minced with a 20 mL filter plunger and passed through a 70 μm cell strainer, followed by rinsing with cold PBS. Splenocytes were collected in a total volume of 20 mL and centrifuged at 300× *g* for 5 min at 4 °C. The supernatant was discarded, and the cell pellet was washed with 10 mL of cold PBS and centrifuged at 300× *g* for 5 min at 4 °C. After washing, the cells were resuspended in fresh growth medium consisting of RPMI-1640 (PanEco, Moscow, Russia), 10% FBS (Cytiva, Marlborough, MA, USA), 2 mM L-glutamine (Gibco, Grand Island, NY, USA), and 1% penicillin/streptomycin (Gibco, NY, USA). The cells were then frozen for long-term storage in liquid nitrogen in a custom cryovial medium containing 50% FBS, 40% RPMI-1640, and 10% DMSO. The medium volume per cryovial was 1 mL, and the cell concentration was 10 million/mL.

For T-cell activation, frozen splenocytes were thawed at 37 °C, washed, and resuspended in OptiVitro^®^ T-cell Serum-free Medium (ExCell Bio, Suzhou, China). Mouse CD3/CD28 T-cell activation beads (Elabscience, Wuhan, China) were added to the cells at a 3:1 ratio (splenocytes:beads), along with 25 IU/mL recombinant hIL-2 (R&D Systems, Abingdon, UK). The cells were cultured in 48-well TC-treated plates (Jet BIOFIL, China) in 500 µL of medium per well with an initial splenocyte concentration of 2 million/mL at 37 °C for three days, after which the cells were washed on a magnetic stand to remove the beads. The bead-free cells were then transfected according to the protocol described in Section 4.9 or stained with antibodies and DARPin according to the protocol described in Section 4.10.

### 4.9. Transfection of Murine T-Cells

Activated murine T-cells prepared according to the protocol described in Section 4.8 were transfected with various mRNA-LNP variants. After activation, cells were seeded into a 96-well U-bottom TC-treated plate (Corning Inc., Corning, NY, USA) at 80,000 cells per well in 100 µL of RPMI-1640 medium (PanEco, Russia), 10% FBS (Cytiva, USA), 2 mM L-glutamine (Gibco, USA), 1% penicillin/streptomycin (Gibco, USA), and 200 IU/mL recombinant hIL-2 (R&D Systems, UK). A total of 750 ng/well mRNA-LNP was added to the cells in 20 µL of buffer. The same volume of buffer without LNPs was added to the untransfected control. After adding mRNA-LNPs, the cell suspension and LNPs were carefully mixed by pipetting. Cytometric analysis was performed 24 h after transfection. All samples were prepared in triplicate.

### 4.10. Flow Cytometry

DARPin binding specificity to the CD8 receptor was assessed on 3-day-activated mouse splenocytes using a BD LSRFortessa flow cytometer. DARPin was preconjugated with the Cy5 fluorochrome (Materials and Methods, Section 4.4). The cell suspension (1 × 10^6^ cells per sample) was preincubated in 300 µL FACS buffer (PBS with 1% FCS and 2 mM EDTA) supplemented with FcR-blocking antibodies (Elabscience^®^, #E-AB-F0997A^®^) for 15 min at 4 °C. The samples were then supplemented with CD3-PE (Elabscience^®^, #E-AB-F1103UD) and CD4-AF488 (Elabscience^®^, #E-AB-F1097L) antibodies. DARPin-Cy5 was added to the test sample at a final concentration of 0.05 µg/mL. The cells were incubated in the dark for 40 min at 4 °C and then washed twice by centrifugation. Before analysis, the samples were supplemented with DAPI (0.1 µg/mL) to exclude dead cells. The specificity of DARPin-Cy5 was assessed by comparing the staining profile and percentage of positive cells with the control antibody CD8-APC.

The ability of LNPs decorated with anti-CD8-DBCO-DARPin to specifically target CD8 lymphocytes was assessed in vitro on mouse splenocytes based on GFP expression. One day after the addition of LNPs, the cells were washed free of medium in PBS without protein, stained with the vital dye FVS780 (BD Horizon™, #565388) for 15 min at 4 °C, and washed with FACS buffer. They were then stained with the following antibodies: CD3–AF700 (BioLegend, #100216), CD4–ElabFluor^®^ Violet 450 (Elabscience^®^, #E-AB-F1353UQ), and CD8–APC (Elabscience^®^, #E-AB-F1104UE) for 30 min at 4 °C, followed by washing with excess FACS buffer. The analysis was performed on a BD LSRFortessa flow cytometer.

### 4.11. Statistical Analysis

All data are presented as means ± SD, as described in the figure captions. One-way ANOVA followed by Šidák’s multiple comparisons test was used to compare differences between three or more independent samples. All statistical analyses were performed with GraphPad Prism 9.3.1 (GraphPad Software Inc., San Diego, CA, USA). Statistical significance was defined as *p* < 0.05. Statistical tests for significance are described in the individual figure legends. All results were obtained from at least three biological replicates unless otherwise specified in the figure legends.

## Figures and Tables

**Table 1:** Physicochemical characteristic of investigated LNP-5 and LNP-5@IPAD-1 with different IPAD-1-to-PEG molar ratio (0.10:1.5 and 0.27:1.33 mol%) (*n* = 6).

Sample	Z Average, nm	PDI, A.U.	Particles/mL	ζ-Potential, mV	EE, %
LNP-5 (no DARPin, PBS)	84.5 ± 1.24	0.161	4.49 × 10^12^	+5.4 ± 0.77	>99
LNP-5 + IPAD-1 (0.10 mol%, PBS)	79.9 ± 0.45	0.112	3.02 × 10^12^	−10.6 ± 0.77	91
LNP-5 + IPAD-1 (0.10 mol%, Tris)	82.6 ± 0.76	0.103	3.46 × 10^12^	−19.7 ± 0.66	90
LNP-5 + IPAD-1 (0.27 mol%, PBS)	84.2 ± 0.32	0.186	2.25 × 10^12^	−15.2 ± 1.43	97
LNP-5 + IPAD-1 (0.27 mol%, Tris)	108.5 ± 1.32	0.239	3.42 × 10^12^	−20.0 ± 2.14	91

## Data Availability

The original contributions presented in this study are included in the article and Supplementary Materials. Further inquiries can be directed to the corresponding authors.

## Supplementary Materials

The following supporting information can be downloaded at: https://www.mdpi.com/article/10.3390/ijms27177755/s1.

## Abbreviations

Cys	Cysteine
DARPin-Cys-Mal	Anti-CD8 DARPin conjugated via a maleimide–thiol bond to DMG-PEG-2000-Mal
DARPin-pAzF	DARPin anti mCD8 with pAzF
DARPins	Designed Ankyrin Repeat Proteins
DBCO	Dibenzocyclooctyne
hEKL	Human enteropeptidase light chain
IPAD-1	DARPin anti mCD8 conjugate with DSPE-PEG(2000)-DBCO
LNPs	Lipid nanoparticles
Lys	Lysine
pAzF	p-azido-L-phenylalanine
PEG	Polyethylene glycol
PylRS	Pyrrolysyl tRNA synthetase
SPAAC	Copper-free azide-alkyl cycloaddition
TRX-A	Thioredoxin A
