# Supplementary material for: Genetic Code Expansion Enables Oriented, Site-Specific Conjugation of DARPins to Lipid Nanoparticles for Selective mRNA Delivery to CD8+ T Cells
Source: Int J Mol Sci. 2026 Aug 29;27(17):7755. doi: 10.3390/ijms27177755 (PMC13565844; doi:10.3390/ijms27177755)
Supplement: Supplementary file 1 [file ijms-27-07755-s001.zip › ijms-4525194-supplementary.pdf]

Supplementary Table S1 – Plasmid constructs used in this study

Name	Antibiotic	Origin	ORIF1		ORIF2	
			**Promoter**	**Gene**	**Promoter**	**Gene**
pHpyl-pAzFPylRS-GlnS	Chloramphenicol	P15A	GlnS	*AzFPylRS*	P_{proK}	*tRNA*^{Pyl}
pET30a__His10_DDDDK_DARPin_mCD8_GG-ncAA-GS	Kanamycin	F1	T7	anti-mCD8 DARPin-GG-amber-GS	N/A	N/A
pS_AG_TPL_eGFP_Mod	Ampicillin	ColE1	T7	eGFP	N/A	N/A

Supplementary Figure S1 – Capillary gel electrophoresis analysis of in vitro transcribed eGFP-encoding mRNA. The expected length of the transcript was approximately 1,200 nt. The sample was analyzed using a Qsep system (Bioptic, Changzhou, China) with the 5× Lower Alignment Marker-C109120-100A. The electropherogram (RFU vs. retention time) showed a single predominant peak corresponding to an apparent electrophoretic size of approximately 1,395 nt. No prominent lower-molecular-weight peaks indicative of RNA degradation or truncated transcripts were detected, supporting the high integrity and homogeneity of the mRNA preparation. The reported fragment size represents an apparent electrophoretic size and should not be interpreted as the exact nucleotide length of the modified RNA transcript.

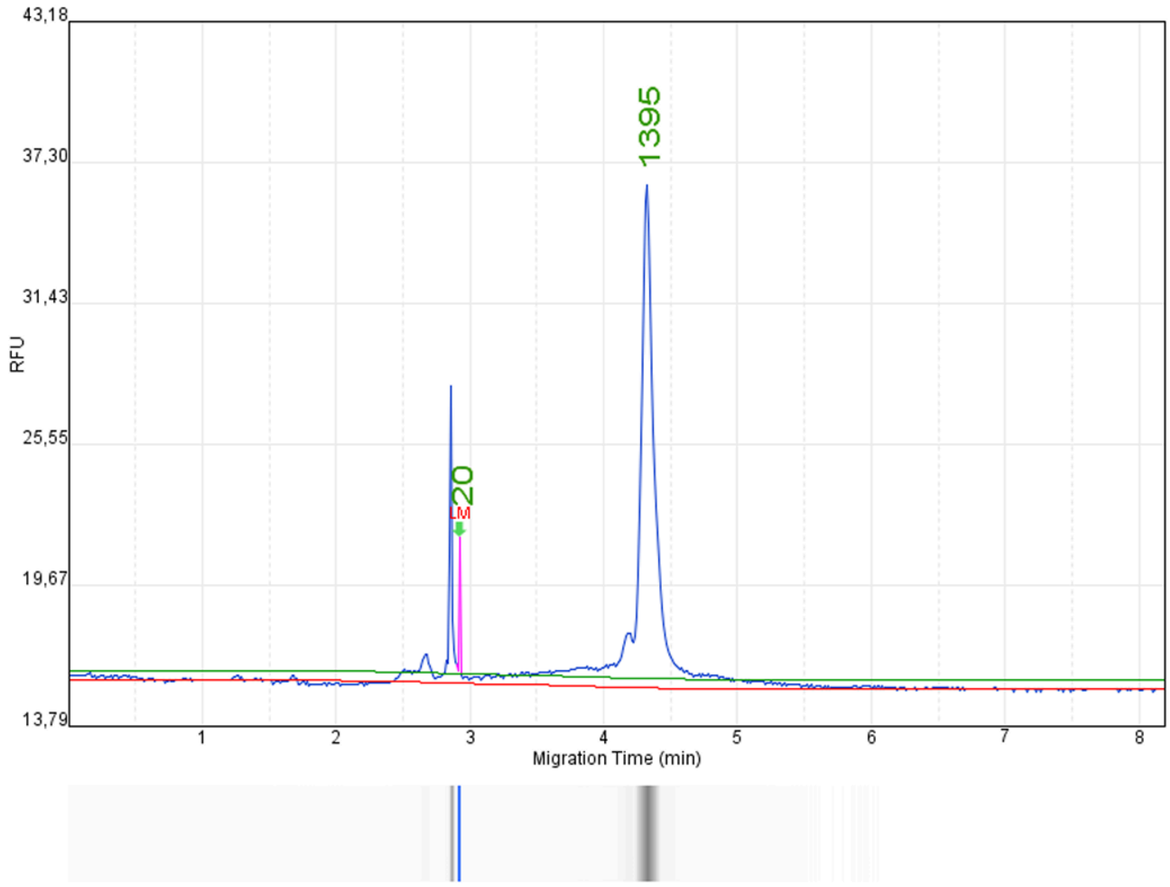

Supplementary Figure S2 - FMO controls for CD4 and CD8 staining. Cells were pre-gated as described in Figure 2C. Dot plots show CD4-AF488 vs. CD8-DARPin-Cy5 expression on live (DAPI^{-}) CD3^{+} T cells. FMO controls lacking either CD4-AF488 or CD8-DARPin-Cy5 confirm the staining specificity of the full panel.

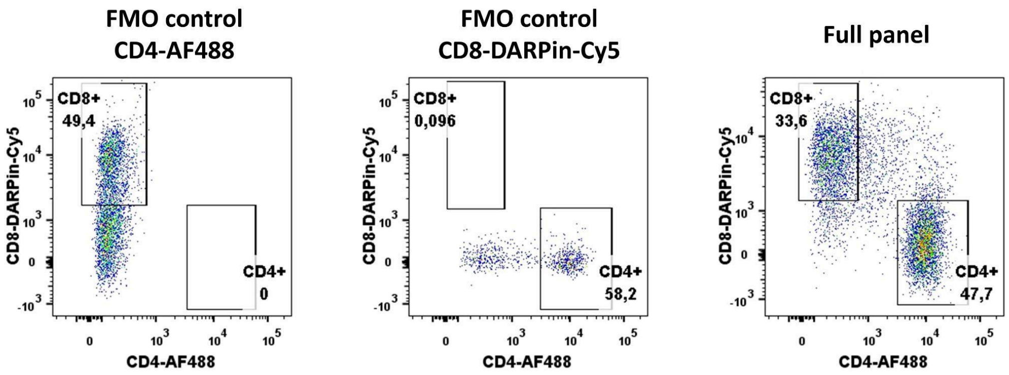

Supplementary Figure S3 - Specificity of CD8-targeted LNP decorated with DARPin via click chemistry. (A) Gating strategy and (B) representative dot plots showing GFP expression in CD3 T-cells and distribution between CD8⁺ and CD4⁺ subsets after treatment. (n=2)

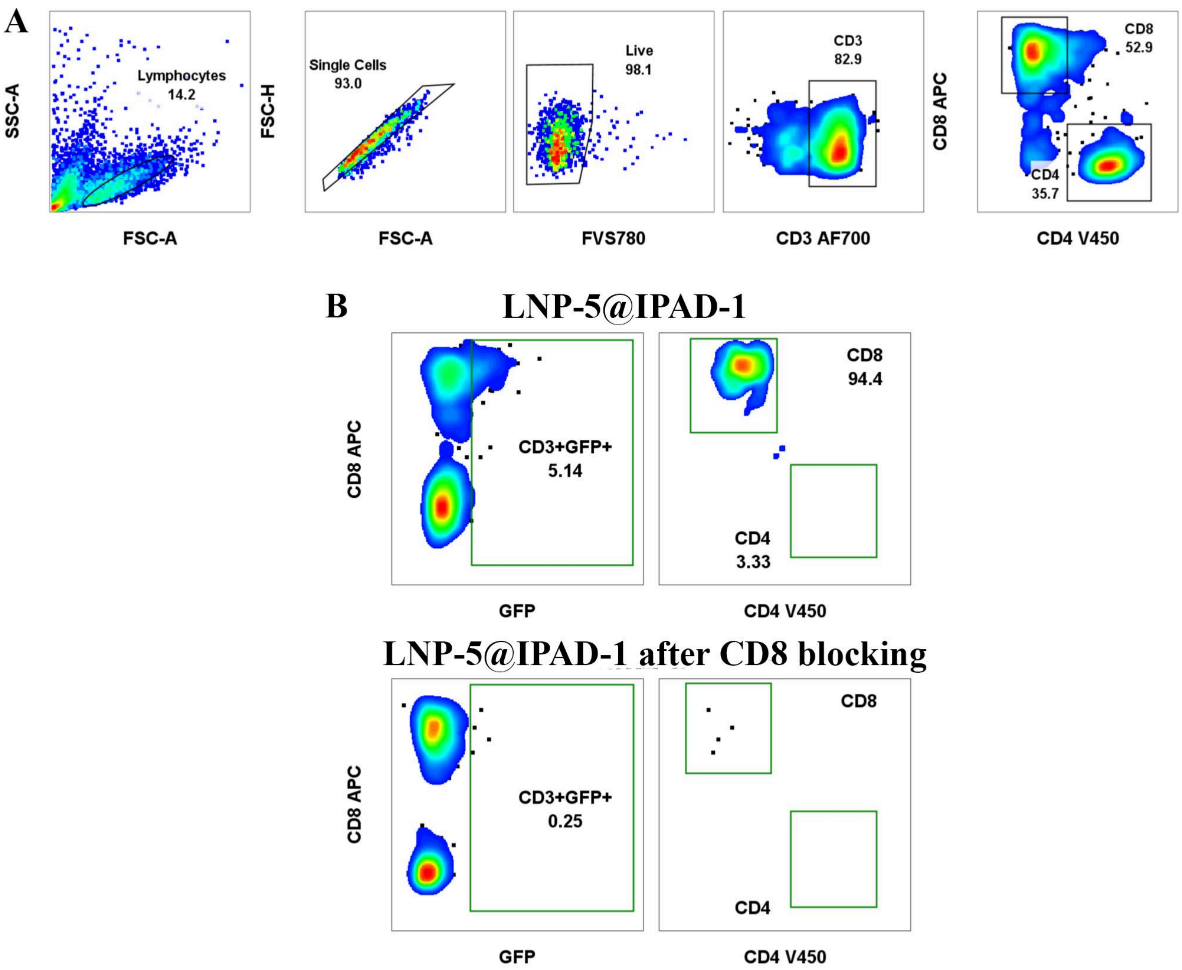

Supplementary Table S2. Physicochemical characteristic of investigated LNP-5, LNP-5-Mal and LNP-5-Mal with DARPin-Cys decoration (n=6)

**Sample (DMG-PEG-2000-Mal content, mol%)**	Z average, nm	PDI, A.U.	ζ-potential, mV
Initial LNP-5 (0) PBS	84.5 ± 1.24	0.16	+ 5.4 ± 0.77
LNP-5-Mal (0.27) PBS	85.9 ± 0.90	0.10	- 4.6 ± 0.20
LNP-5-Mal (0.27) + DARPin-Cys PBS	90.8 ± 1.60	0.10	- 8.7 ± 1.30
